# Enrichment and functional characterization of copper-binding peptides from food hydrolysates

**DOI:** 10.1039/d5ra05166e

**Published:** 2025-08-28

**Authors:** Rebeca L. Fernandez, Vanessa J. Lee, Samuel E. Janisse, Justin J. O'Sullivan, Amanda Caceres, Marie C. Heffern

**Affiliations:** a Department of Chemistry, University of California, Davis One Shields Avenue Davis CA 95616 USA mcheffern@ucdavis.edu

## Abstract

Imbalances in cellular copper are increasingly implicated in metabolic disorders. Food-derived peptides are gaining attention for their ability to alleviate metabolic disease symptoms with little to no toxicity. In this work, we enriched copper-binding peptides from enzymatic digestions of rice bran protein hydrolysates *via* Cu(ii)-based immobilized-metal affinity-based separations, identified the sequences by mass spectrometry, and performed physicochemical and sequence analysis of the enriched peptides. A subset of the enriched peptides representing a range of sequences and hydrophobicities were synthesized and individually assessed further for their solution-based Cu(ii) reactivity and cellular activity. Among the peptides tested, Cu(ii)-binding was most potent with those containing a histidine as the second residue, reminiscent of pseudo-ATCUN motifs. Relative Cu(ii)-binding affinity showed good correlation with the ability of the peptides to reduce Cu(ii) reactivity, as measured with a reaction-based probe, while discrepancies in trends were observed in the scavenging activity of Cu(ii)-generated ROS. To test the hypothesis that solution-based Cu(ii)-binding properties predict cellular bioactivity, we assessed whether the individual Cu(ii)-binding peptides could recapitulate the previously reported AMPK activation in hepatocytes by whole rice bran hydrolysates. This represents a critical validation step for using *in vitro* metal-binding assays to predict biological function. While the majority of the peptides affected AMPK activation, the degree and direction varied in ways that Cu(ii) chelation could not explain, suggesting that the beneficial behavior of the hydrolysates cannot be simplified to its *in vitro* Cu(ii) reactivity. Nonetheless, we demonstrate a strategy for identifying putative metal-binding peptides from complex food-derived mixtures and characterizing their chemical and biological activity.

## Abstract 2

Copper-binding peptides are enriched from rice bran protein hydrolysates and characterized for copper-associated function.

## Graphical abstract

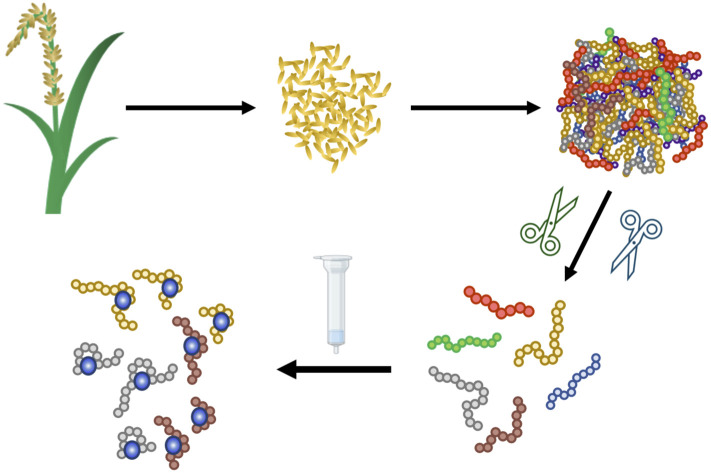

## Introduction

Copper plays essential roles in a host of biological processes such as antioxidant defense, immune response, and glucose metabolism.^1,2^ Its imbalance is implicated in metabolic disorders *via* pathological mechanisms that include protein aggregation, reactive oxygen species (ROS) generation, and mitochondrial disruption.^3,4^ Recent reports have suggested potential connections between therapeutic agents for these metabolic disease states and copper-binding activity.^5–8^ Natural and synthetic copper chelators are under active evaluation for their ability to alleviate diabetic symptoms given that elevated copper levels have been reported in the tissues and serum of diabetic patients. In contrast, as copper deficiency has been found in relation to metabolic syndrome and obesity-related disorders, copper ionophores have also been investigated as potential candidates for treating these conditions. A notable example of the potential relationship between copper and diabetes is in the ability of the antidiabetic agent, metformin, which primarily acts by activating AMP-activated protein kinase pathway (AMPK), to alter mitochondrial copper and even form stable complexes with the Cu(ii) ion.^9,10^ Understanding the copper-modulating behavior of molecules that alleviate symptoms of metabolic disorders may help clarify the mechanisms by which the two could be connected and open new strategies for therapeutic intervention.

Food rich in fruits, vegetables, and grains are associated with lower incidence of disorders like metabolic syndrome, cardiovascular disease, cancer, and diabetes.^11–13^ Dissecting the components of such foods and their corresponding bioactivities may thus bring to light functional molecules with potential therapeutic activities, low toxicity, and good oral bioavailability. In this study, we sought to establish a workflow for isolating Cu(ii)-binding peptides from food-derived hydrolysates and characterizing them at the molecular and cellular level, using rice bran as a model food source. Extracts and oils derived from rice bran, the major byproduct of the rice processing industry, have been gaining commercial popularity for their medicinal and cosmetic benefits combined with low cost. Among its potential health benefits, rice bran protein hydrolysates (RBPH) – a mixture of bioactive peptides obtained from extraction, protein isolation, and digestion of rice bran – have been shown to alleviate conditions associated with metabolic disorders, including lipid- and cholesterol-lowering activities.^14,15^

It has been suggested that the health benefits of RBPH are derived from their ability to quench ROS and chelate metals.^10,12^ Kubglomsong *et al.* has indeed shown that hydrolysates of the albumin component of rice bran can chelate copper. The study was performed in the context of identifying peptides that can inhibit the copper-dependent enzyme, tyrosinase.^16^ In analyzing peptide fractions separated by reverse-phase high-performance liquid chromatography (RP-HPLC), both copper chelation and tyrosinase inhibition decreased with increasing retention time suggesting a relationship between the two activities. A similar dependence of copper chelation on RP-HPLC retention time has been observed in studies of chickpea and sunflower protein hydrolysates.^17–19^ Whether copper-chelating peptides are present beyond the albumin component of RBPH has yet to be determined. Additionally, the specific peptides that contribute to the metabolic health benefits of RBPH, and whether their activity relates to their copper-binding effects, have yet to be identified. These gaps may be due to the complex nature of the hydrolysate mixture that complicates the discovery of copper-binding, bioactive components.

In this work we aimed to identify copper-interacting components within complex RBPH mixtures, characterize their chemical reactivity, and assess their potential cellular bioactivity. We reduced the complexity of our mixture by enriching for copper-binding components *via* a copper-loaded immobilized metal affinity chromatography (IMAC) column approach.^20^ The individual peptides in the enriched fractions were identified and sequenced *via* mass spectrometry-based methods, and assessed for sequence-specific and physicochemical characteristics; then, a subset of the peptides were synthesized for subsequent evaluation. The synthesized peptides were tested and compared for their ability to chelate copper, affect Cu(ii) reactivity, and scavenge Cu(ii)-generated ROS. Interestingly, we found that Cu(ii)-binding activity was not predictive of the strength of antioxidant activity. We also characterized the ability of the individual peptides to perturb copper metabolism and shift AMPK levels in the hepatic cell line, HepG2. While the majority of the peptides affected AMPK activation, the effects varied both with potency and direction and did not correlate to Cu(ii)-associated solution chemistry. Surprisingly, most of the peptides tested had minimal impact on the copper trafficking markers we tested, and the most potent peptide in this respect did not correspond to the most reactive species in our solution-based studies. The lack of correlations points to the likely importance of other factors, such as cellular uptake and/or protein targets in peptide bioactivity. Though copper-binding alone is not predictive of bioactivity, our work nonetheless provides a platform by which functional food-derived peptides with copper-associated bioactivity may be identified and tested.

## Results and discussion

### Enrichment and identification of copper-binding RBPH peptides

RBPH peptide populations were obtained from commercial sources of rice bran *via* two methods of enzymatic digestion. The two approaches were employed both to mimic products that result from human physiological digestion and to assess the copper-binding properties of peptides generated *via* different digestion conditions. The first digestion condition incubated rice bran with pepsin in acidic conditions and was followed by trypsin digestion in slightly basic conditions. Pepsin, found in the stomach, has promiscuous cleavage at aromatic and hydrophobic residues. Conversely, trypsin is specific to cleaving C-terminal to arginine and lysine.^21^ The second digestion scheme used papain, an enzyme with broad specificity found in papaya that cleaves preferentially at the C-terminus of basic or hydrophobic residues. In addition to this method mimicking human digestion, pepsin/trypsin (Pep/Tryp) yielded chemically distinct products than the papain digestion. The efficiencies of the enzyme digestions were assessed *via* quantitation of the presence of free amines.^22,23^ As compared to a leucine standard, the Pep/Tryp digestion showed a slightly higher degree of hydrolysis than the papain method (Fig. S1).

**Fig. 1:**
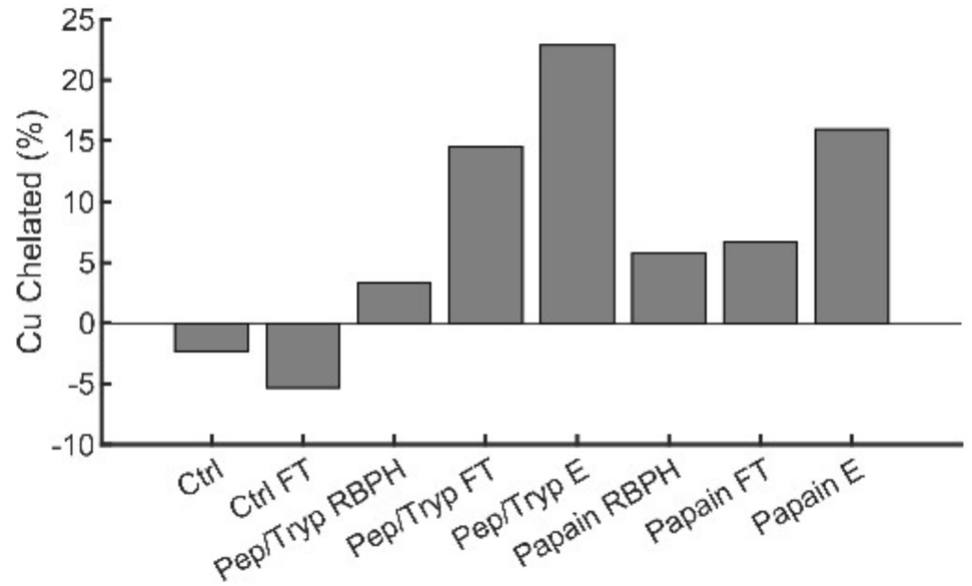
The copper chelating capacity of hydrolysate mixtures, digested with either a pepsin/trypsin scheme (Pep/Tryp) or papain, was assessed *via* a colorimetric Cu(ii) chelator, zincon. The absorption of the zincon–Cu(ii) complex at 615 nm in the presence of 10 μg of hydrolysate mixture, 200 μM zincon, and 100 μM CuSO_4_ was monitored. The percent chelation is reported for the rice bran hydrolysate mixture prior to enrichment (RBPH), the flow through (FT, *i.e.* what is not retained on the Cu(ii)–IMAC resin), and the enriched fraction (E, *i.e.* what is retained on the Cu(ii)–IMAC resin and eluted with low pH). The mixtures are compared to a solution of CuSO_4_ (Ctrl) and the same solution of CuSO_4_ applied to the Cu(ii)–IMAC resin (Ctrl FT). The increase in chelated copper upon incubation with the Pep/Tryp elution mixture suggests that proteins hydrolyzed with different digestion methods have distinct copper-binding properties.

To enrich peptides with copper-binding propensities, the hydrolysates were applied to a Cu(ii)-iminodiacetic acid IMAC resin. Retained peptides can be eluted either *via* application of a competing ligand or by pH modulation. We opted to apply the latter over the former to facilitate the study of the eluents with downstream assays; the use of the competing ligand would require an additional purification step to remove the ligand from the mixture as the presence of such species can otherwise interfere with assays focused on copper-binding properties. To confirm that the enriched RBPH peptide fractions could bind Cu(ii) in solution, the fractions were assessed for their ability to outcompete Cu(ii) from the colorimetric chelator, zincon (Fig. 1). This competition assay relies on monitoring the changes in absorption of the zincon–Cu(ii) complex, which has a known *K*_d_ of 4.68 × 10^−17^ M,^24–26^ and the data is reported as the percentage of copper chelated (see Materials and methods). In the case of both enzymatic digestion schemes, we note that the IMAC-enriched subpopulations (Pep/Tryp E and papain E, where E refers to eluent) registered a higher amount of chelated copper than the hydrolysate mixtures prior to enrichment (Pep/Tryp RBPH and papain RBPH). Additionally, in both digestion schemes the “flow-through” (FT) fractions (*i.e.* subpopulations that were not retained on the resin on initial application; Pep/Tryp FT and papain FT) registered a lower amount of chelated copper than the eluted hydrolysate mixtures. Interestingly, the copper-binding abilities of the hydrolysate mixtures and their enriched fractions were influenced by the digestion method, with the Pep/Tryp RBPH exhibiting a lower percentage of chelated Cu(ii) than papain RBPH. Upon enrichment, this trend was reversed and the Pep/Tryp E chelated more Cu(ii) than the papain E.

We subsequently identified the distinct components in the enriched subpopulations *via* mass spectrometry (MS). Samples were desalted and separated in the microflow regime (50 μL min^−1^) on a C18 column connected online to an Orbitrap-HF mass spectrometer. MS2 spectra were acquired over the course of the LC run in data-dependent acquisition mode and searched against the UP000059680 (*Oryza sativa*) FASTA database. Over 100 peptides per digestion were identified (see SI Table 1). To the best of our knowledge, this is the first time that RBPH subpopulations have been enriched based on copper-binding abilities, and that the complete sequences of Cu(ii)–IMAC-enriched peptides from food hydrolysates have been identified and sequenced.

IMAC enrichment has demonstrated potential to discover metal-binding species that have evaded classical discovery and are difficult to predict based on sequence information alone.^20,27–29^ Importantly, the separation of metal-binding species *via* this technique can be used to gauge the relative strength of chelators in a complex biological matrix. However, the typical IMAC workflow leaves the precise metal coordination site ambiguous. In addition, strong metal chelators may “strip” the metal from the chelating resin and elute in the flow through, evading detection. Thus, enrichment of rice bran hydrolysate peptides *via* IMAC may prevent the identification of the most high-affinity copper coordinating peptides. It is likely that in many cases strong copper-binding would be a result of well-characterized motifs, like the amino terminal copper-binding motif (ATCUN) and derivatives.^30–32^

### Amino acid compositional analysis reveals selective enrichment characteristics

To characterize the peptide populations retained by Cu(ii)–IMAC enrichment, we analyzed the amino acid composition, physicochemical properties, and positional distribution of the enriched fractions from both digestion schemes. This comprehensive analysis focused exclusively on peptides identified after the IMAC enrichment step to understand the selectivity and architectural preferences of theCu(ii)-loaded resin.

**Fig. 2:**
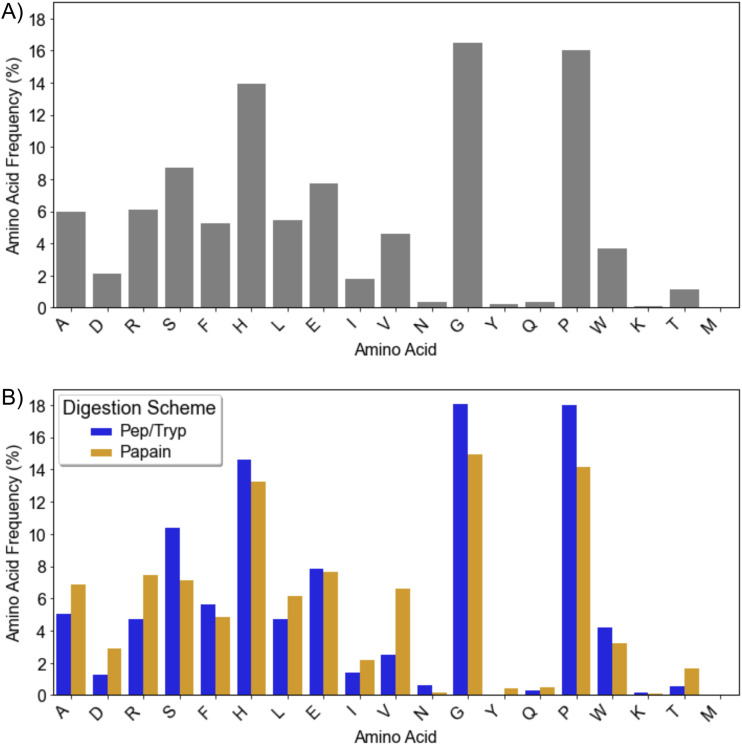
Cu(ii)–IMAC enrichment demonstrates selective retention of peptides with specific compositional signatures. Amino acid frequencies represent the percentage of each amino acid among all amino acid residues in the peptide population. (A) Global amino acid composition of all Cu(ii)^+^–IMAC enriched peptides shows dramatic enrichment of glycine, proline, and histidine compared to typical rice bran protein profiles. (B) Amino acid composition patterns differ Pep/Tryp and papain digestion methods.

Cu(ii)–IMAC enrichment demonstrated selectivity for peptides with specific physicochemical signatures. The enriched populations shows length distributions spanning 5–30+ amino acids with a predominant broad peak around 10–25 amino acids, isoelectric points primarily centered around neutral pH (pI ∼ 7) with smaller populations extending to more basic ranges, and net charges predominantly between +1 to −2 (Fig. S2). The range of net charges and isoelectric points centered around neutral pH (pI ∼ 7) indicate that retention is driven by specific copper coordination chemistry rather than simple electrostatic attraction to the resin matrix. Additionally, this charge distribution does not show a bias for highly basic sequences, suggesting that Cu(ii)–IMAC selects for peptides with optimal metal-coordinating architectures. Amino acid compositional analysis revealed dramatic alterations compared to typical rice bran protein profiles (Fig. 2A). Glycine and proline emerged as the most highly enriched residues, each comprising 16–18% of the total amino acid content, representing substantial enrichment from their typical abundances of 6–7% and 8–10%,^33^ respectively, in rice bran proteins. As might be expected from IMAC enrichment, histidine contributed a remarkable increase from its typical 1–3% abundance^34^ in native rice bran proteins, being present in 13–15% to the enriched populations.

Despite undergoing identical enrichment procedures, peptides derived from papain and Pep/Tryp digestion exhibited statistically significant differences in their physicochemical properties (Fig. S3 and S4). The Kolmogorov–Smirnov test revealed significant differences in length distribution patterns (*p* = 0.0016), indicating that enzymatic specificity influences which peptide populations become accessible for Cu(ii)–IMAC retention. While statistically significant differences were detected in isoelectric point, hydrophobicity, and net charge distributions due to large sample sizes, the practical differences between digestion methods were modest. Both digestion methods showed similar pI distributions centered around 7. Pep/Tryp-enriched populations exhibited an additional notable pI peak around 8–9 and demonstrated higher proportions of positively charged peptides (+1, +2), consistent with trypsin's cleavage specificity after basic residues.

**Fig. 3:**
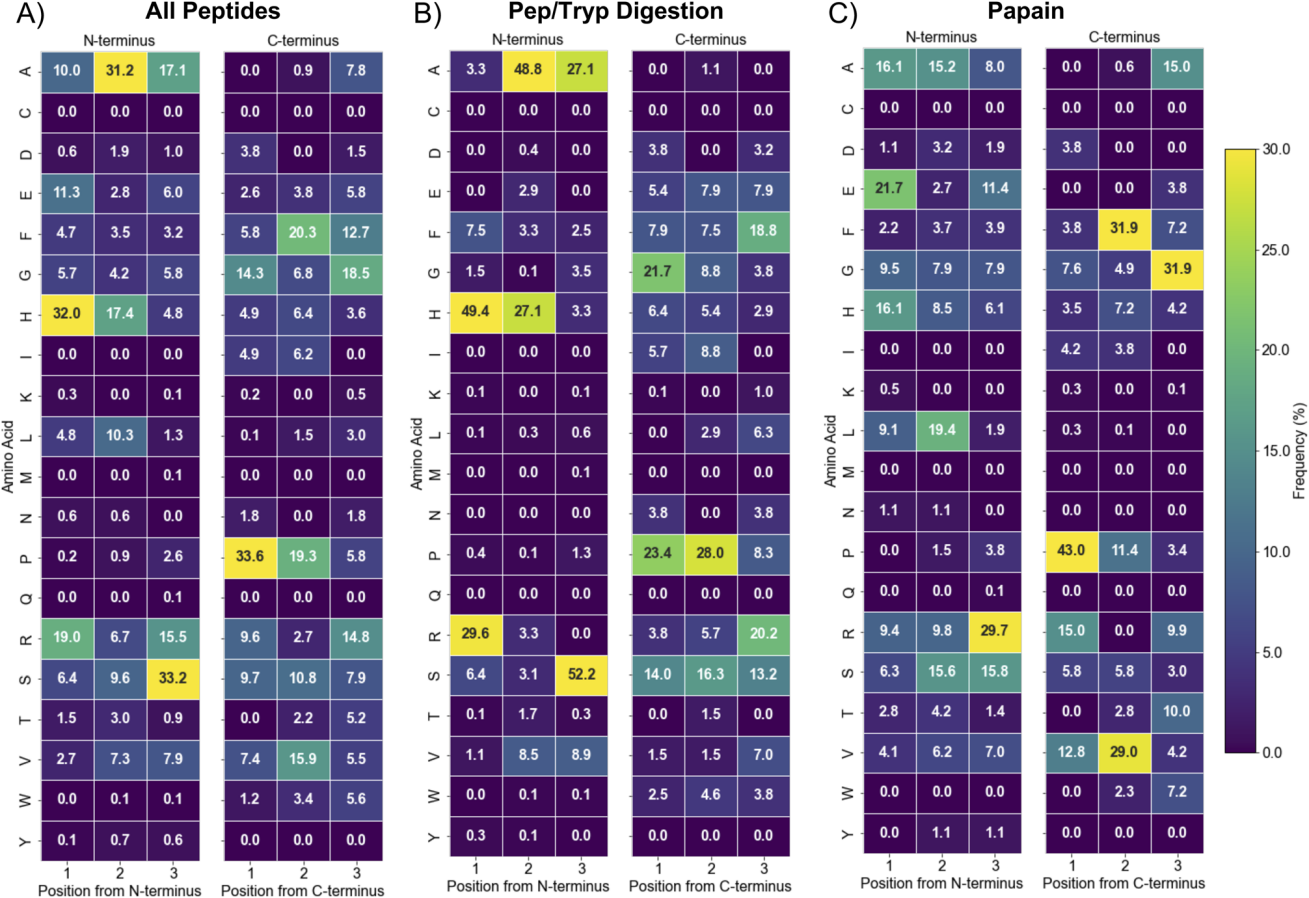
Position-specific amino acid frequency analysis reveals spatial organization preferences in Cu(ii)–IMAC enriched peptides. Heat maps show amino acid frequencies (%) at N-terminal (positions 1–3) and C-terminal (positions 3rd-last to last) positions for (A) all enriched peptides, (B) Pep/Tryp-digested peptides, and (C) papain-digested peptides. Notable features include dramatic N-terminal histidine enrichment (32% overall, 49% in Pep/Tryp *vs.* 16% in papain), C-terminal arginine positioning, and digestion-specific patterns that reflect enzymatic cleavage specificity.

Positional analysis revealed biases in spatial organization underlying the compositional patterns (Fig. 3), demonstrating that Cu(ii)–IMAC enrichment selects for specific peptide architectures rather than simple amino acid abundance. Most remarkably, while histidine comprises only 13–15% of total amino acids globally (Fig. 2), 32% of all enriched peptides contains histidine at the N-terminal position—a striking example of selective enrichment for optimal metal-coordinating architecture. Additional positional hotspots include alanine at N-terminal position 2 (31.2% in this position, *versus* 5–7% total) and serine at N-terminal position 3 (33.2% in this position, *versus* 7–10% total). Proline, which makes up 15–18% of the total amino acid population, shows a biased localization to the third-to-last residue from C-terminus of the enriched peptides (33.6%). Interestingly, glycine displays a contrasting pattern: while making up 16–18% of total amino acids, it shows marked positional exclusion at the N-terminus (only 4.2–5.8% across the three N-terminal positions) and variable C-terminal representation (6.8–15.5%). This suggests that while glycine contributes to overall peptide flexibility and may facilitate metal coordination through backbone interactions, its placement at key terminal positions may be less favorable for optimal metal-binding architecture. The contrast of these positional abundances *versus* the overall compositional analysis highlights that spatial arrangement, not just abundance, drives retention.

Digestion method profoundly influenced these spatial patterns (Fig. 3B and C). Pep/Tryp digestion creates peptides with dramatically enhanced N-terminal histidine positioning (49.4% *vs.* 16.1% for papain in position 1, 27.1% *vs.* 8.5% for papain in position 2) and strong alanine enrichment at position 2 (48.8% *vs.* 15.2% for papain). Papain digestion shows distinct patterns including enhanced arginine at N-terminal position 3 (29.7% *vs.* 0% for Pep/Tryp) and notable C-terminal enrichment of glycine (31.9% *vs.* 3.8% in Pep/Tryp), phenylalanine and valine in the second-to-last C-terminal position (31.9% *vs.* 7.5% for Pep/Tryp and 29% *vs.* 1.5% for Pep/Tryp, respectively). Notably, proline's C-terminal abundance remained consistently high across both digestion methods, mirroring the pattern observed in all enriched peptides and suggesting its importance in overall enrichment rather than digestion-specific architecture. While digestion methods show some differences in amino acid compositions (Fig. 2B), the positional distributions reveal much more dramatic architectural differences, with Pep/Tryp generating more predictable coordination site placement while papain's broader specificity produces more variable distribution of coordinating residues.

While Fig. 2 shows the dramatic overall enrichment of key amino acids like glycine, proline, and histidine compared to typical rice bran proteins, the positional analysis reveals that their spatial organization, not just their presence, fundamentally influences the peptide populations accessible for metal affinity enrichment. These findings demonstrate that Cu(ii)–IMAC selectivity operates through architectural recognition, explaining why simple compositional analysis alone cannot predict metal-binding capacity.

Having characterized the enrichment selectivity and architectural features of the Cu(ii)–IMAC retained populations, we next sought to validate these findings through individual peptide analysis. One disadvantage of the workflow is that it produces very low yields of copper-enriched RBPH (200 g rice bran to ∼2 mg enriched hydrolysate); we thus opted to continue our studies by selecting, synthesizing, and studying a subset of peptides from those identified in our analysis. From the enriched peptides, we identified conserved core sequences that appear across the enriched populations (SI Table 1). Nine peptides representing core sequences that appear in both digestion schemes were selected for further biochemical and cellular analysis. The peptides were selected to cover a range of represented amino acids and hydrophobicities/retention times. These peptides were synthesized by solid-phase peptide synthesis and verified *via* ESI-MS (Fig. S5). Peptides were grouped and named with a number and letter identifier based on common core sequences (series 1 contains ASEGG, series 2 contains HWPLPPF, series 3 contains VPSGHPI) and length (series A are 7-mers, series B are 8-mers, series C are 9-mers) (Table 1). Peptide hydrophobicity was predicted *via* the method of Kyte and Doolittle to calculate the grand average of hydropathy index (GRAVY);^35^ a negative GRAVY value represents a peptide's hydrophobicity, a positive value indicates a more hydrophilic peptide, and the magnitude of the score suggests the extent of a peptide's hydropathy. The GRAVY predictions were compared to the relative experimental hydrophobicity of the peptides as assessed using RP-HPLC mass spectrometry (HPLC-MS) wherein longer elution times indicate increased hydrophobicity. Intriguingly, the GRAVY scores do not agree with the order of hydrophobicity obtained by HPLC-MS retention time. While the GRAVY score predicts series 1 as the most hydrophilic, series 3 as the most hydrophobic, and series 2 in between, the retention times suggests series 2 as the most hydrophobic. As the GRAVY-score-based prediction only considers the amino acid composition and length, but not the sequence, this discrepancy points to the importance of considering the peptide structure and configuration in its hydropathy, and not just size and elemental composition. This finding is of note, as traditional analysis of food hydrolysate mixtures focuses on percent amino acid composition, and in some cases sequence motifs.

**Table 1:** Peptide sequences selected among the identified components of the Cu(ii)–IMAC-enriched RBPH

Identifier	Peptide sequence	GRAVY score^a^	Elution time^b^ (min)
1A	RHASEGG	−1.57	1.98
1B	ASEGGHG	−0.99	2.26
1C	RHASEGGHG	−1.62	1.89
2A	HWPLPPF	−0.33	65.71
2B	PHWPLPPF	−0.49	65.71
2C	GPHWPLPPF	−0.48	65.71
3A	VPSGHPI	0.26	30.20
3B	VVPSGHPI	0.66	33.57
3C	FVVPSGHPI	0.90	55.99

a The grand average of hydropathy index (GRAVY) score predicts a larger positive value to be a more hydrophilic peptide.

b Elution times are reported from reverse-phase HPLC-MS as experimental measures of hydrophobicity.

### Characterization of static and reactive copper-binding by individual rice bran-derived peptides

**Fig. 4:**
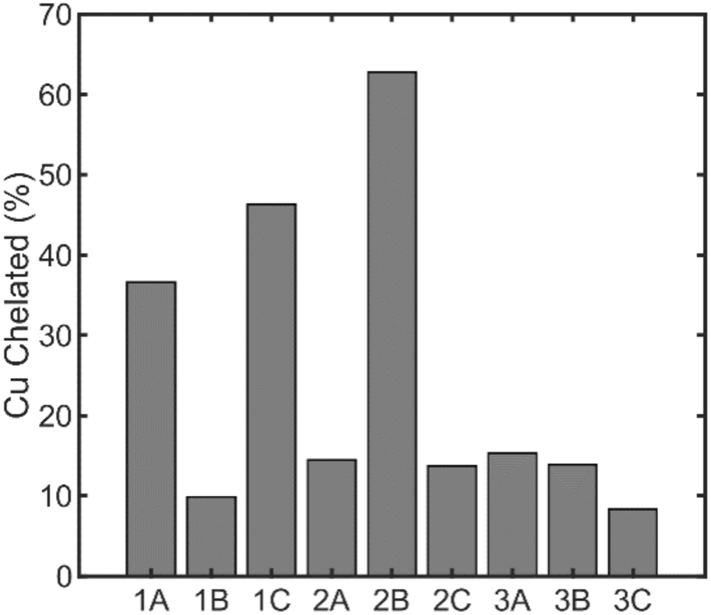
Nine rice bran-derived peptides were assayed for solution-based copper coordination. Cu(ii) chelating capacities of the peptides were measured by monitoring the absorption of the zincon–Cu(ii) complex at 615 nm in the presence of 10 μg of peptide, 200 μM zincon, and 100 μM CuSO_4_.

**Fig. 5:**
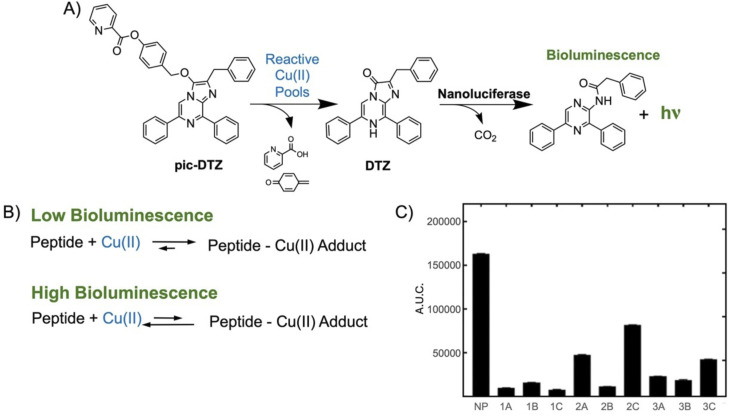
(A) The picolinic ester-caged diphenylterazine (pic-DTZ) is a Cu(ii)-responsive caged imidazopyrazinone probe that generates diphenylterazine (DTZ) upon ester hydrolysis by Cu(ii). In the presence of nanoluciferase (Nluc), DTZ is enzymatically converted to produce light. (B) The pic-DTZ-derived signal can be applied to probe the degree by which rice bran derived peptides can affect “free” Cu(ii) in solution. A peptide that can chelate or decrease the reactivity of Cu(ii) will retain more pic-DTZ and less DTZ in solution, resulting in low bioluminescence signal. In contrast, a peptide that has less interaction with Cu(ii) will maintain the “free” and reactive form of Cu(ii) in solution, resulting in high bioluminescence signal. (C) Calculated area under the curve (AUC) of luminescence (integrated over 20 minutes) of 1 μM pic-DTZ, a Cu(ii)-responsive probe, in the presence of 10 μM CuSO_4_ with no peptide (NP) or with pre-incubated solutions of 10 μM CuSO_4_ and 100 μM peptide. Error bars shown as SEM, *n* = 3.

The Cu(ii)-binding capacities of the synthesized peptides were individually assessed using the zincon competition assay described above. All peptides exhibited higher Cu(ii)-binding capacity than the whole hydrolysate mixtures (≥10% copper chelated), with peptides 1A, 1C, and 2B chelating the largest percentage (Fig. 4). We also tested the ability of the peptides to interact with Cu(ii) using a bioluminescence assay developed by our group that uses a pro-substrate for the bioluminescent NanoLuc enzyme termed pic-DTZ.^36^ The pic-DTZ pro-substrate contains a picolinate ester that masks the imidazopyrazinone substrate, DTZ, from interacting with NanoLuc, inhibiting light production. Binding of Cu(ii) at the picolinate group induces hydrolysis and self-immolative cleavage of the picolinate ester mask, revealing the active substrate and resulting in light emission in the presence of NanoLuc (Fig. 5A). In this way, this system can be used to assay the impact of biomolecules specifically on reactive pools of Cu(ii), or “free” Cu(ii) (Fig. 5B).^36^ Lower light output (reported in terms of area-under-the-curve, AUC) of a kinetic enzyme assay relative to a control solution of CuSO_4_ salt with no peptide (NP), corresponds to a peptide's ability to reduce the available reactive Cu(ii) pools in solution, either by chelation or reduction (Fig. 5C). All peptides induce a reduction in light output relative to NP, confirming their chelating capacities. In agreement with the data from the zincon–Cu(ii) competition study, we observe the most reduction in signal from peptides 1A, 1C, and 2B, validating that these peptides chelate the highest percentage of Cu(ii) of the peptides tested. While the rest of their sequences differ, peptides 1A, 1C, and 2B share the feature of histidine as the N-terminal second residue; an amino acid sequence known to feature high affinity Cu(ii) coordination.^31,37–40^ The finding that this feature is predictive of Cu(ii) binding is in agreement with the extensive body of literature characterizing pseudo-ATCUN motifs (where histidine is in the second rather than third position) and related N-terminal copper-binding sequences. This literature highlights the importance that small variations in the motif sequences can destabilize copper coordination. In comparing the zincon and pic-DTZ assays, we note that the copper–peptide mixtures with the least chelated Cu(ii) (1B and 3C, Fig. 4), are not the solutions with the most reactive Cu(ii) (2A and 2C, Fig. 5C), suggesting alternative mechanisms for affecting Cu(ii) reactivity besides chelation.

### Probing the effects of copper-binding rice bran hydrolysate peptides on ROS levels

**Fig. 6:**
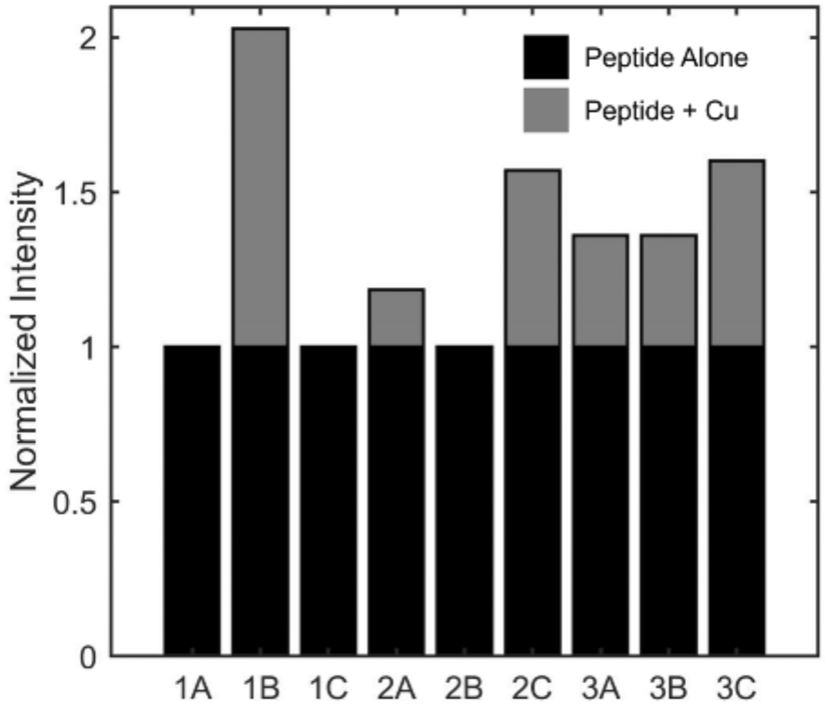
Fluorescence-based assay *via* conversion of 3-CCA to 7-OH-CCA (ex. 338 nm, em. 450 nm) allows the relative measurement of redox protection from Cu(ii)-induced HO˙ generation by RBPH peptides (40 μM) in the absence (black bar) or presence of 10 μM CuSO_4_ (grey bar). Data shown are normalized to peptide in the absence of Cu(ii) with further controls reported in Fig. S6.

A prominent mechanism by which aberrant levels or miscompartmentalization of copper induces cellular and tissue damage is *via* its production of ROS *via* Fenton-like chemistry.^41^ We investigated the ability of the RBPH peptides to influence Cu(ii)-induced ROS levels. We employed a fluorescent assay that monitors, by proxy, the formation of the hydroxyl radical (HO˙) generated *in situ via* the reduction of Cu(ii) by ascorbic acid. In this assay, nonfluorescent coumarin-3 carboxylic acid (3-CCA) is oxidized to fluorescent 7-OH-CCA in the presence of HO˙ radicals; a decrease in fluorescence thus corresponds to a change in signal associated with Cu(ii)-induced ROS availability.^42^ We evaluated the effects of the peptides on the production of fluorescent 7-OH-CCA in the absence or presence of added Cu(ii) (Fig. 6 and S6). Peptides 1A, 1C, and 2B, which were shown to have the highest chelation capacity and lowest reactivity to pic-DTZ, were found to produce the least amount of 7-OH-CCA in the presence of added Cu(ii). The decreased fluorescence could be a result of the protective effects of these peptides on ROS production, due to their tight copper coordination and related ability to reduce reactive Cu(ii). Alternatively, a change in fluorescence could result from Cu(ii)–peptide complexes scavenging formed ROS species prior to their interaction with the probe. Interestingly, the fluorescence output associated with the remaining peptide samples (*i.e.* not 1A, 1C, and 2B), does not directly trend with their copper chelation capacity nor effects on reactive Cu(ii). These findings suggest that additional factors beyond the scope of this study, like potential interactions with trace metal ions or other Fenton-type chemistry, may influence the abilities of these peptides to interact with ROS.

### Assessing the cellular activity of copper-binding rice bran hydrolysate peptides

**Fig. 7:**
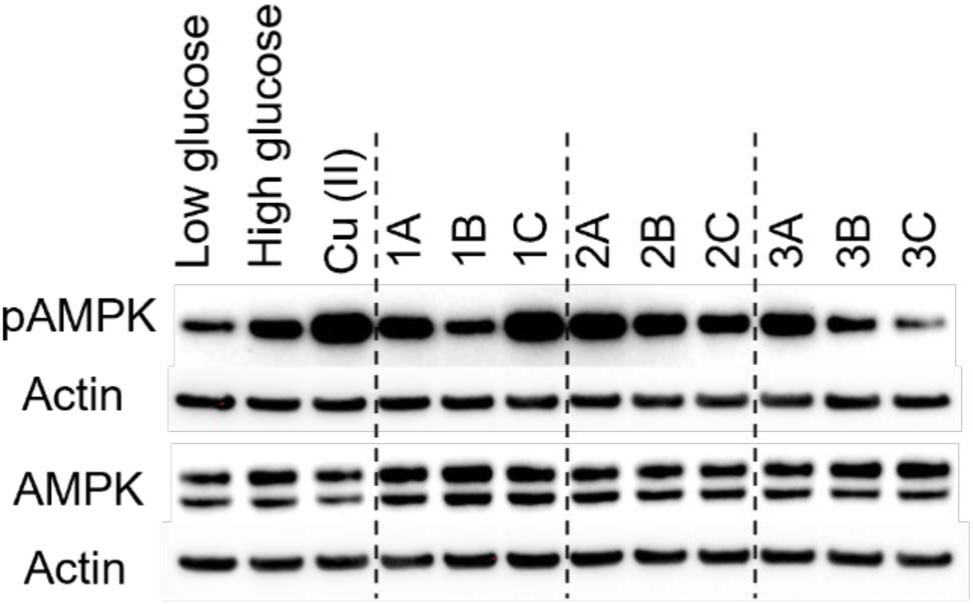
Representative western blot images of phosphorylated AMPK (pAMPK) and AMPK proteins. Insulin-resistant HepG2 cells were treated with a low and high glucose control, 10 μM CuSO_4_, or 10 μM rice bran derived peptide and incubated for 24 h. Cell lysates were collected and protein was visualized using antibodies specific for pAMPK and AMPK with β-actin as a control. Densitometry is been included in Fig. S7.

The addition of rice bran hydrolysates to hepatocytes exposed to high glucose has been reported to induce AMPK phosphorylation (*i.e.* can activate AMPK) and insulin resistance.^43^ Copper bioavailability and AMPK activation have also been linked,^44–47^ but we note that whether copper bioavailability correlates with increased or decreased AMPK phosphorylation depends on both cell type and conditions. As the selected peptides were derived from a pool of hydrolysates enriched by copper coordination, we probed whether a relationship exists between AMPK activation and the copper-binding effects of the isolated RBPH peptides. We tested the RBPH peptides in a well-established insulin-resistant HepG2 cell model,^43,48^ as this model has previously been used to monitor AMPK phosphorylation and exhibits cellular ROS injury, which is typically associated with copper dyshomeostasis. HepG2 cells were exposed to simultaneous high glucose levels and serum starvation,^43^ and the effects of subsequent treatment with the individual RBPH peptides under sustained high-glucose exposure were evaluated. After a 24 hour treatment, cells were lysed, pelleted, and the abundances of pAMPK and AMPK were visualized *via* western blot (Fig. 7 and S7). Protein levels of peptide treated samples in high glucose exposure were compared to subsequent low-glucose exposure in the absence of peptides, high-glucose exposure in the absence of peptides, and high-glucose exposure in the presence of Cu(ii) salt.

Under the conditions tested, relative to the sustained high-glucose exposure, subsequent exposure to low glucose decreases AMPK phosphorylation, while the addition of Cu(ii) with high glucose exposure leads to an increase in pAMPK levels. Interestingly, the individual peptides show varying degrees of AMPK activation, even within each series, despite the sequence similarities in a given numerical series. When comparing peptides within series 1, 1A and 1C induce higher pAMPK levels as compared to 1B, which is consistent with the higher chelating abilities of the former two. However, such a correlation is not observed with series 2: while 2B showed the highest chelation capacity, 2A induces elevated AMPK phosphorylation. In contrast to the series 1 and 2 peptides, series 3 peptides, most notably 3C, induce a decrease in pAMPK.

**Fig. 8:**
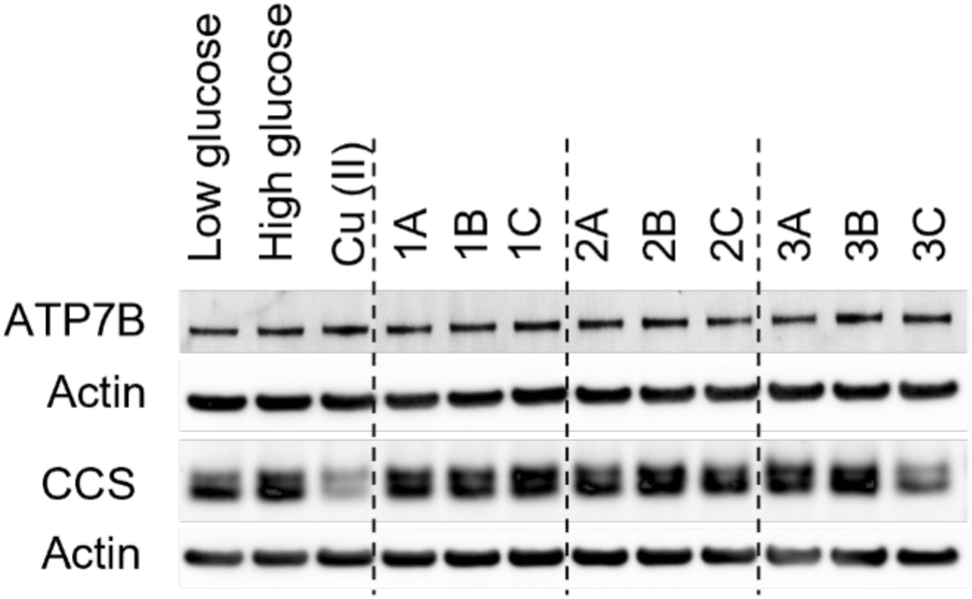
Representative western blot images of ATP7B and CCS proteins. Insulin-resistant HepG2 cells were treated with a low and high glucose control, 10 μM CuSO_4_, or 10 μM rice bran derived peptide and incubated for 24 h. Cell lysates were collected and protein was visualized using antibodies specific for ATP7B and CCS with β-actin as a control. Densitometry has been included in Fig. S8.

We next tested if either the solution-based behavior of the peptides or the effects of the peptides on AMPK activation could be related to peptide effects on cellular copper status. We probed two intracellular copper markers upon peptide treatment: the copper-transporting P-type ATPase (ATP7B) and the copper marker chaperone for superoxide dismutase (CCS) (Fig. 8 and S8). Typically, an increase in ATP7B suggests elevated intracellular copper levels,^24^ while an increase in CCS is ascribed to a decrease in intracellular copper and ROS.^25,26^ The bands corresponding to ATP7B did not show changes across treatment conditions, suggesting that the cells are not in a regime in which there is an alteration in copper transport. Relative to high glucose, CCS expression is slightly reduced with low glucose exposure, while the addition of Cu(ii) under high glucose conditions results in a significant reduction in expression. While most of the peptides show no significant impact on CCS expression, a decrease in the levels of CCS relative to the high glucose control is observed upon treatment with 3C. This was surprising, as 3C exhibits low copper-binding affinity and low ROS scavenging abilities. Interestingly, 3C also shows the lowest AMPK activation, but as to whether these two observations are linked will require further investigation. Importantly, solution-based copper-binding affinity was not predictive of AMPK activation patterns, revealing a critical limitation of *in vitro* metal-binding assays for predicting peptide bioactivity.

Taken together, despite the peptides displaying high copper reactivity in solution, we find that the solution-based reactivity trends do not translate to the cellular context, neither with AMPK activation nor in perturbing cellular copper status. Additionally, while the peptides affected AMPK activation, the trends could not be correlated to cellular copper status, pointing to copper-independent mechanisms for this process. Other factors may contribute to the bioactivities of the peptide, including whether the peptides are internalized,^49^ their antioxidant properties,^50^ and cellular cytokine production.^43,51^ It may also be the case that the bioavailable copper in the cell culturing conditions we applied are not sufficient for peptide interactions to occur. Finally, while we tested individual peptides, it is possible that the activity of rice bran hydrolysates involves cooperative or opposing interactions between peptides in the mixture. These results demonstrate the complexity of peptide bioactivity, where factors beyond copper-binding—including cellular uptake, membrane permeability, and intracellular targets—likely determine biological effects. This disconnect highlights important limitations in using solution-based metal-binding assays to predict cellular function and has important implications for nutraceutical development, where metal-chelating properties are often used as bioactivity predictors.

## Conclusion

Food-derived peptides, including those from rice bran hydrolysates, hold rich potential as functional therapeutics and nutraceuticals. While whole hydrolysates have promising health benefits, factors associated with their preparation and processing, such as the enzymatic hydrolysis procedure, can affect their bioactivity, introduce inconsistencies, and hinder commercial development from low yields.^52,53^ We indeed observed that the digestion scheme can affect the copper-binding capacities of the resulting hydrolysates. Identifying and separating components of the hydrolysates that carry the desired functions may open opportunities for new therapeutic developments, but the complexity of these mixtures makes it challenging to ascribe activity to individual components. Importantly, tying functional motifs to specific solution-based properties of hydrolysate mixtures or peptides, will facilitate the development of therapeutics. In this work, we aimed to identify components of RBPH that have copper-associated function by first focusing on peptide populations with copper-binding propensities *via* IMAC-based enrichment. To the best of our knowledge, this was the first effort to specifically sequence copper-binding peptides from complete rice bran hydrolysates and identify sequence-specific trends in enriched populations and differences in digestion schemes. The enrichment approach allowed us to identify, synthesize, characterize, and compare individual peptides from RBPH for their copper-binding behavior in solution. We tested for chelation capacity *via* a competitive chelator approach and found that both enriched hydrolysates and the individual peptides tested showed higher chelation effects than whole hydrolysates. These results highlight the potential for fraction-based enrichment or applying individual components as a means to improve bioactivity of food-derived products. This approach could facilitate the development of standardized, peptide-based nutraceuticals with defined copper-modulating properties, addressing current challenges in food hydrolysate commercialization where batch-to-batch variability limits therapeutic applications. Copper chelation capacities of the peptide were compared to Cu(ii) reactivity (as measured by Cu(ii)-dependent hydrolysis) and Cu(ii)-dependent ROS levels (as measured by Cu(ii)/ascorbate-induced hydroxyl radical generation). While histidine content has been previously shown to affect Cu(ii)-binding activity, our comparison revealed the importance of histidine positioning as well: the most potent Cu(ii)-associated activities across all the assays were observed in peptides with histidines in the second position. We interestingly observed discrepancies in trends between Cu(ii) reactivity and ROS levels, thus pointing to the need to consider these two behaviors separately in assessing copper-binding function.

Several limitations of our approach should be acknowledged. The IMAC enrichment may miss the highest-affinity copper-binding peptides that strip copper from the resin, and our cellular studies were limited to a single hepatocyte model without assessment of peptide internalization or membrane permeability. Additionally, the disconnect between solution-based copper-binding and cellular bioactivity suggests that other factors—including peptide stability, cellular uptake, and protein–protein interactions—play crucial roles that warrant further investigation. Identifying individual peptides in the mixture enabled us to assess whether copper-binding components of RBPH could be directly related to previously reported cellular function, activation of the AMPK signaling cascade. Interestingly, most of the peptides affected AMPK signaling, but the directionality and magnitude of the effects varied. The trends could not be ascribed to solution-based Cu(ii) chemistry of the peptides, suggesting that either the pathway effects are copper-independent or require consideration of other factors such as membrane permeability. While we did not find copper-binding to be predictive of effects on AMPK signaling, our strategy of enriching and identifying hydrolysate peptides with a bias for copper-binding activity opens future opportunities to identify whether other bioactivities of RBPHs directly relate to their copper chelation capacities. Finally, our approach offers a framework that is applicable to other nutrient-derived peptides for assessing how metal-interacting abilities might relate to their beneficial effects in cellular and *in vivo* models. Future studies examining peptide behavior in enterocyte models and assessing membrane permeability would provide valuable insights into the oral bioavailability and first-pass metabolism of these copper-binding peptides.

## Materials and methods

### Chemicals and reagents

Commercially available rice bran was obtained from NOW Foods. Papain, trypsin, pepsin, 2,4,6-trinitrobenzenesulfonic acid (TNBSA), CuSO_4_ were purchased from Millipore Sigma (St. Louis, MO). Phosphate buffered saline (PBS), trifluoroacetic acid (TFA), ethylenediaminetetraacetic acid (EDTA), and all solvents were obtained from Fisher Scientific (Waltham, MA). DSC-18 SPE tubes were purchased from Millipore Sigma (St. Louis, MO).

### Rice bran protein extraction and hydrolysate preparation

Rice bran was defatted with 1 : 3 w/v of rice bran to hexanes three times for 30 minutes each. The mixture was centrifuged at 4000*g* at 25 °C for 30 minutes. The supernatant was discarded, and the precipitate dried overnight.

Dried defatted rice bran protein was stored in aluminum foil bags at −20 °C. A mixture of 1 : 4 w/v rice bran to nanopure water was prepared, after which the pH was adjusted to 9.5 with 1 M NaOH. The mixture was stirred at 500 rpm for 45 minutes at room temperature. The mixture was centrifuged at 15 000*g* for 30 minutes. The pH of the supernatant was adjusted to 4.5 with 1 M HCl and centrifuged at 15 000*g* for 30 minutes. The precipitate was collected, freeze dried, and stored at −20 °C.

Rice bran protein extracts (RBPE) were prepared in nanopure water at 8 mg mL^−1^. For hydrolysis with pepsin and trypsin, 1 : 100 w/w pepsin was added to the RBPE solution, and the pH was adjusted to pH 1.5. The solution was shaken at 37 °C for 120 minutes and then neutralized with 1 M NaOH to stop digestion. 1 : 100 w/w trypsin was added to the mixture and incubated at 37 °C for 120 minutes. The solution was heated at 95 °C for 10 minutes to stop digestion. The mixture was centrifuged at 3000 rpm for 10 minutes. The supernatant included the rice bran protein hydrolysates (RBPH) which was freeze dried and stored at −20 °C. For hydrolysis with papain, 1 : 100 w/w enzyme was added to the protein extracts and incubated at 37 °C. The pH was adjusted to pH 8 with 1 M NaOH, and the solution was incubated at 37 °C for 30 minutes. The enzymatic hydrolysis was stopped by placing the solution in boiling water for 5 minutes followed by an ice bath. The protein hydrolysates were freeze-dried and stored at −20 °C.

### Degree of hydrolysis

2,4,6-Trinitrobenzenesulfonic acid (TNBSA) was used to assess the degree of hydrolysis after enzyme digestion. 0.1% (v/v) TNBSA solution in nanopure water was prepared. 2 mg of each sample was prepared in 1 mL of 10 mg per mL sodium dodecyl sulfate (SDS). 125 μL of each sample, with water as a control, was added to 1 mL of sodium phosphate buffer (250 mM, pH 8.5) and 1 mL of TNBSA. Leucine was used as a control (0–5 mM standards). Solutions were incubated at 50 °C in the dark for 1 hour. 2 mL of 100 mM HCl were added to stop the reaction. Solutions were allowed to sit for 30 minutes before absorbance measurements at 340 nm. The degree of hydrolysis was calculated as follows:DH% = (*h*/*h*_tot_) × 100%where *h* is the hydrolysis of each sample as compared to the standard curve and *h*_tot_ is the total molar equivalence per gram of protein.

### Copper-based enrichment *via* immobilized metal affinity chromatography

In a centrifuge tube, 1 mL of Profinity IDA IMAC resin (Promega, Madison, WA) was washed with three column volumes of nanopure water. Then, 2 mL of 1 M CuSO_4_ was added to the column to form IDA–Cu(ii) resin and washed with 3 column volumes of nanopure water. Three column volumes of PBS, pH 7.4, was used to equilibrate the column. 25 mg of rice bran protein hydrolysates (RBPH) in 4 mL PBS were added to the column and incubated for 1 hour after which the unbound protein hydrolysates were collected. The column was washed with 30 column volumes of PBS before elution with 500 mM acetic acid.

### LC-MS/MS of copper-binding rice bran hydrolysates

Dried peptides were reconstituted in nanopure water and quantified *via* a peptide fluorescence assay (Pierce). 5 μg of peptide were analyzed by microflow liquid chromatography connected online to the mass spectrometer (Ultimate 3000 and Q Exactive HF). Peptides were loaded onto a 1 mm × 150 mm PepMap C18 column and separated at a flow rate of 50 μL min^−1^ with a gradient ranging from 3% acetonitrile to 80% acetonitrile over 80 min, a ramp to 95% for 4 min, an isocratic hold at 95% for 4 min, followed by a ramp down to 3% over 4 min, and finally a hold at 3% for 28 min. MS1 and MS2 data were acquired on Xcalibur (Thermo Fisher Scientific). MS spray voltage was set to 4 kV; capillary temperature set to 320 °C; sheath, auxiliary, and spare gas maintained at 35, 5, and 0, respectively; and S-lens set to 40. MS1 spectra were collected at a resolution of 60 000 with a AGC target of 3 × 10^6^ and a max ion injection time of 50 ms. The mass range of MS1 was 250–2000 Da. MS2 spectra were collected using data-dependent acquisition (DDA) with the top 12 ions from the MS1 scan being selected for fragmentation with dynamic exclusion set to 15 s. The normalized collision energy was set to 28%. For MS2 spectra, the AGC was set to 1 × 10^5^ with a maximum ion injection time of 86 ms and spectra were acquired at a resolution of 15 000.

### Identification of hydrolyzed rice bran peptides

Raw LC-MS/MS files were searched using MSFragger in FragPipe.^54,55^ Spectra were searched against the Uniprot protein database UP000059680 (*Oryza sativa*) concatenated with the reverse protein sequences (decoys) and common contaminates. A “closed” search was conducted with a precursor tolerance set to −1.2 and 1.2 Da and fragment mass tolerance set to 20 ppm. Digestion constrains were set to “non-specific” and maximum of two missed cleavages with cysteine methionine oxidation (+15.994900), pyro-glutamic acid or loss of ammonia at the peptide N-terminus (−17.0265), N-terminal acetylation (+42.0106), and loss of water on glutamate on the peptide N-terminus (−18.0106) as variable modifications. The reverse-decoy method was used to estimate FDR. Peptide (PSM) and protein FDR was set to 0.01.

### Peptide sequence analysis and statistical comparisons

Peptide sequences identified from LC-MS/MS were analyzed using custom Python scripts with pandas, NumPy, and peptides libraries. Physicochemical properties including peptide length, isoelectric point, hydrophobicity (GRAVY score), and net charge were calculated for each sequence. Global amino acid composition was determined for all enriched peptides and stratified by digestion method. Position-specific amino acid frequencies were analyzed using 3-amino acid windows at N-terminal (positions 1–3) and C-terminal (3rd-last to last) regions. Statistical comparisons between digestion methods used Mann–Whitney *U* and Kolmogorov–Smirnov tests with significance set at *p* < 0.05. Data visualization employed matplotlib and seaborn with consistent color schemes (orange for papain, blue for Pep/Tryp). Position-specific frequencies were visualized as heat maps using the viridis colormap.

### Identification of putative copper binding peptide sequences

Peptide identifications from the pepsin/trypsin and papain digestion experiment were imported into Python. Peptides with a probability less than 0.99 were removed and sequence motifs were generated *via* custom Python script. For each peptide identified, a section of length *N*, where *N* was required to be equal to or less than the total length of the peptide, was added to a list. For peptides with lengths greater than *N*, sequences were recorded iteratively from the N-terminus till the C-terminus was contained in the sequence motif. The generated motif list was then filtered based on frequency and the most frequent motifs were compared across digestion conditions using the normalized Hamming distance.^56^

### Copper chelation assessment

Copper chelating capacity was assessed using a colorimetric copper(ii) chelator, zincon. 10 μg of each hydrolysate or peptide sample, determined by the Pierce quantitative fluorometric peptide assay (Thermo Fisher, Waltham, MA), was added to each well in a 96-well plate. Stock solutions of 2 mM zincon, and 2 mM CuSO_4_ were prepared. Final concentrations in each well were 200 μM zincon and 100 μM CuSO_4_. Each sample in PBS, pH 7.4 was allowed to incubate with the CuSO_4_ for 10 minutes at 37 °C before addition of zincon. The zincon–copper complex absorbs at 615 nm at pH 7.4. The copper chelating percent was calculated as follows:Copper chelating% = [(*A*_control_ − *A*_sample_)/*A*_control_] × 100%where *A*_sample_ is the absorbance of the sample and copper subtracted from the absorbance of the sample, copper, and chelator.

### Peptide synthesis and purification

Peptides were synthesized at a 0.2 mmol scale either by hand or by microwave assistance with standard Fmoc solid-phase peptide synthesis (SPPS) methodologies. In the case of unassisted synthesis, coupling reagents, 2-(1*H*-benzotriazol-1-yl)-1,1,3,3-tetramethyluronium hexafluorophosphate (HBTU) and *N*,*N*-diisopropylethylamine (DIEA), were used in 4 times molar excess for each coupling. Wang resin was shaken in 3 times the volume of *N*,*N*-dimethylformamide (DMF) to swell the resin. The resin was end-capped using 50 times the concentration of acetic anhydride and pyridine in DMF. Between each step of the synthesis, the resin was washed three times with DMF; after each coupling, the resin was washed three times with DMF, methanol, and dichloromethane. All amino acids were Fmoc-protected and were coupled in 4 times molar excess to the resin at 95 °C for 20 minutes. A Kaiser test was performed after each coupling to confirm completion. Amino acids were Fmoc deprotected using 3 times volume of 25% piperidine in DMF at 95 °C for 5 minutes.

In the case of assisted synthesis, a CEM Liberty Blue 2.0 automated microwave peptide synthesizer was used. Peptides were synthesized at 0.2 mmol scales using preloaded Wang resin. Synthesis began with a 20 min resin swelling step, after which the amino acid was deprotected. Amino acid couplings were performed using CEM-optimized microwave synthesis conditions: activating reagents diisopropylcarbodiimide (DIC) and OxymaPure, 1 min deprotection at 90 °C, followed by a 2 min coupling at 90 °C. After coupling of the last amino acid, the final step of the synthesis was the deprotection of the N-terminal residue. Upon completion, the peptide-coupled resin mixture was removed from the peptide synthesizer and washed with DMF (10×), DCM (10×), dried for 1–2 hours, and refrigerated at −20 °C.

After synthesis, peptides were cleaved in a trifluoroacetic acid cocktail containing 1.5 g phenol, 1 mL water, 0.5 mL triisopropyl silane, 1 mL thioanisole, and 16 mL trifluoroacetic acid. The solution was poured into chilled diethyl ether to precipitate, then the suspension was centrifuged at 3900 rpm for 10 minutes at 4 °C. The supernatant was decanted, and the pellet was washed again with chilled diethyl ether followed by centrifugation after each wash (3×). The pellet was dried under a stream of nitrogen and refrigerated at −20 °C until further purification.

Purification was performed with RP-HPLC on an Agilent Technologies 1260 Infinity II HPLC with coupled Agilent Technologies 1260 Infinity II UV-vis detection system. Purification of crude peptides were performed using an Atlantis T3 C18 Prep column at a flow rate of 17.06 mL min^−1^ using a gradient of water with 0.1% FA and acetonitrile with 0.1% FA. Fractions containing peptide were confirmed by electrospray ionization mass spectrometry (ESI-MS) using an Agilent Technologies 1260 Infinity II coupled with an Agilent Technologies InfinityLab LC/MSD, after which they were dried and stored at −20 °C.

### pic-DTZ luminescence assay

The assay was conducted using the procedure outlined in O'Sullivan *et al.*^36^ In brief, the wells of a white, opaque, flat-bottom 96-well plate contained 100 μM peptide, 10 μM CuSO_4_, 15 μm pic-DTZ, and 60 nM rNluc (Promega, Nano-Glo Assay Kit). After the addition of pic-DTZ to all wells *via* a multi-channel pipette, the bioluminescent signal was immediately measured at 37 °C for 1 hour using a Molecular Devices SpectraMax i3x plate reader (Molecular Devices, San Jose, CA). GraphPad Prism software was used to calculate the area under the curve of luminescence, integrated over 20 minutes.

### Protective effects against ROS production using 3-CCA

Levels of generated ˙OH in solution were measured as previously described.^42^ A stock solution of 2.5 mM 3-coumarin carboxylic acid and 500 μM ascorbic acid was prepared in 10 mM phosphate buffer, pH 7.4. 50 μL of the CCA/ascorbic acid solution was added to 200 μL of pre-incubated peptide/CuSO_4_ solutions at the described concentrations in 10 mM phosphate buffer, pH 7.4. Time-dependent fluorescence intensity measurements were recorded with excitation at 388 nm and emission at 450 nm over the course of 90 minutes using a Spectramax i3x microplate reader.

### Cell culture and treatment

HepG2 cells were maintained in 1 g per L DMEM media supplemented with 10% fetal bovine serum, 1% sodium pyruvate, 1% glutamine, and 1% penicillin–streptomycin (referred to as complete media). Low glucose media contained low glucose DMEM (1 g per L glucose) supplemented with 10% fetal bovine serum, 1% sodium pyruvate, 1% glutamine, and 1% penicillin–streptomycin. High glucose media was composed of high glucose DMEM (10 g per L glucose) supplemented with 10% fetal bovine serum, 1% sodium pyruvate, 1% glutamine, and 1% penicillin–streptomycin. Serum starvation media was composed of high glucose DMEM (10 g per L glucose), 1% sodium pyruvate, 1% glutamine, and 1% penicillin–streptomycin (referred to as high glucose starvation media).

Cells were incubated at 37 °C with 5% CO_2_ in complete media until 70% confluent. To initiate an experiment, cells were plated at 300 000 cells per well in 6-well plates and allowed to grow for 24 hours. To induce insulin resistance, media was replaced with high glucose starvation media for 18 hours. Cells were treated with low glucose media or high glucose media and supplemented with the RBPH peptides for 24 hours. After treatment, cells were washed with cold PBS, then chemically lysed in RIPA buffer (150 mM NaCl, 1% NP-40, 0.5% sodium deoxycholate, 0.1% SDS, 50 mM Tris–HCl pH 7.4). Each 10 mLs of RIPA buffer contained one EDTA-free protease inhibitor (Roche) and one phosphatase inhibitor tablet (Pierce). Lysates were vortexed, incubated on ice for 30 minutes, then cleared by centrifugation at 15 000*g* for one hour at 4 °C. Lysates were first frozen at −80 °C, after which total protein content was quantified using a bicinchoninic (BCA) assay (Pierce BCA Protein Assay, Thermo Fisher).

### Western blot analysis

After total protein quantitation, samples composed of 10 μg of total protein were prepared with 2-mercaptoethanol (Bio-Rad), PBS, and LDS sample buffer (Invitrogen). Samples were loaded onto a 4–12% Bis–Tris 15-well gel (Bolt, Invitrogen), run in MES SDS buffer (Bolt, Invitrogen) at 100 V for one hour, and then transferred onto a low fluorescence PVDF membrane (1704274, Bio-Rad) using the turbo setting on the Trans-Blot Transfer System (Bio-Rad). Membranes were blocked in 5% bovine serum albumin (BSA) in Tris-buffered saline with Tween-20 solution (TBST, Cell Signaling Technology) for 1 hour at room temperature, rinsed 3 × 5 min with TBST, and then blotted in primary antibody overnight at 4 °C. In the case of CCS, the membranes were blocked in 5% blotting grade blocker in TBST solution for 1 hour, rinsed 3 × 5 min with TBST, and then blotted in primary antibody overnight at 4 °C. The following day, membranes were washed 3× for 5 min with TBST at room temperature and blotted with a secondary antibody in 5% BSA in TBST. Again, in the case of CCS, secondary antibody was incubated in a 5% blotting grade blocker in TBST solution prior to imaging on a ChemiDoc MP Imager (Bio-Rad).

Primary antibodies include the phosphorylated AMPK antibody (1 : 1000, Phospho-AMPKα (Thr172) (40H9) Rabbit mAb #2535, Cell Signaling Technology) which is directed towards the Thr172 AMPKα phosphorylation site, anti-AMPK (1 : 1000, AMPKα Antibody #2532, Cell Signaling Technology), anti-ATP7B (1 : 1000, ab124973, Abcam), anti-CCS (1 : 2000, sc-55561, Santa Cruz Biotechnology), and anti-β-actin (1 : 5000, 4970S, Cell Signaling Technology). For secondary antibodies, anti-rabbit IgG HRP-conjugated antibody (1 : 2000, 7074S, Cell Signaling Technology) was used for pAMPK, AMPK, and ATP7B, anti-mouse IgG AlexaFluor 800 (1 : 2500, A32789, Thermo Fisher) for CCS, and anti-rabbit IgG AlexaFluor 555 (1 : 2500, A28180, Thermo Fisher) for β-actin. When incubated with HRP-conjugated secondary antibody, membranes were shaken for 3 minutes in Crescendo HRP substrate solution (Millipore) prior to imaging. Images were processed using the Bio-Rad Image Lab software.

For densitometry, two separate gels were run and stained for either total AMPK and β-actin or phosphorylated AMPK and β-actin. Bands were quantified using Image Lab software (Bio-Rad). The ratio of β-actin bands to total AMPK bands was calculated from the first blot, after which the ratio of phosphorylated AMPK to β-actin was calculated from a second blot. The ratio of β-actin normalized phosphorylated AMPK to β-actin normalized total AMPK was then calculated.

## Conflicts of interest

There are no conflicts to declare.

## Supplementary Material

RA-015-D5RA05166E-s001

RA-015-D5RA05166E-s002

## Data Availability

The data supporting this article have been included as part of the SI, including the SI Table 1. Custom Python scripts used for peptide sequence analysis are available from the corresponding author upon reasonable request. Supplementary information is available and contains supporting Fig. S1–S8. See DOI: https://doi.org/10.1039/d5ra05166e.
