# Supplementary material for: Enrichment and functional characterization of copper-binding peptides from food hydrolysates
Source: RSC Adv. 2025 Aug 28;15(37):30697–710. doi: 10.1039/d5ra05166e (PMC12394934; doi:10.1039/d5ra05166e)
Supplement: RA-015-D5RA05166E-s001 [file RA-015-D5RA05166E-s001.pdf]

Supplementary Information (SI) for RSC Advances.
This journal is © The Royal Society of Chemistry 2025

Supplementary Information for

Enrichment and functional characterization of copper-binding peptides from food hydrolysates

Rebeca L. Fernandez,[‡a] Vanessa J. Lee,[‡a] Samuel E. Janisse,[a] Justin J. O'Sullivan,[a] Amanda Caceres,[a] Marie C. Heffern*[a]

[‡] Denotes equal contributions
[a] Department of Chemistry, University of California, Davis, Davis, CA 95616, USA
*Corresponding author
E-mail: mcheffern@ucdavis.edu

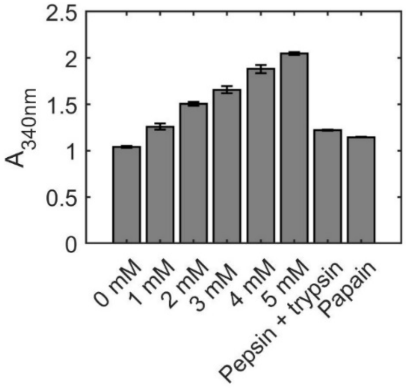

Figure S1. After rice bran peptide mixtures were digested using two distinct methods, pepsin+trypsin and papain, the degree of hydrolysis was assessed. Post digestion, a solution of the hydrolysate mixture and 2,4,6-trinitrobenzenesulfonic acid (TNBS) were reacted and the absorbance of TNBS bound to free amines was read at 340 nm. Comparison to 0-5 mM leucine standards demonstrates that the pepsin/trypsin digestion scheme produced more free amino groups.

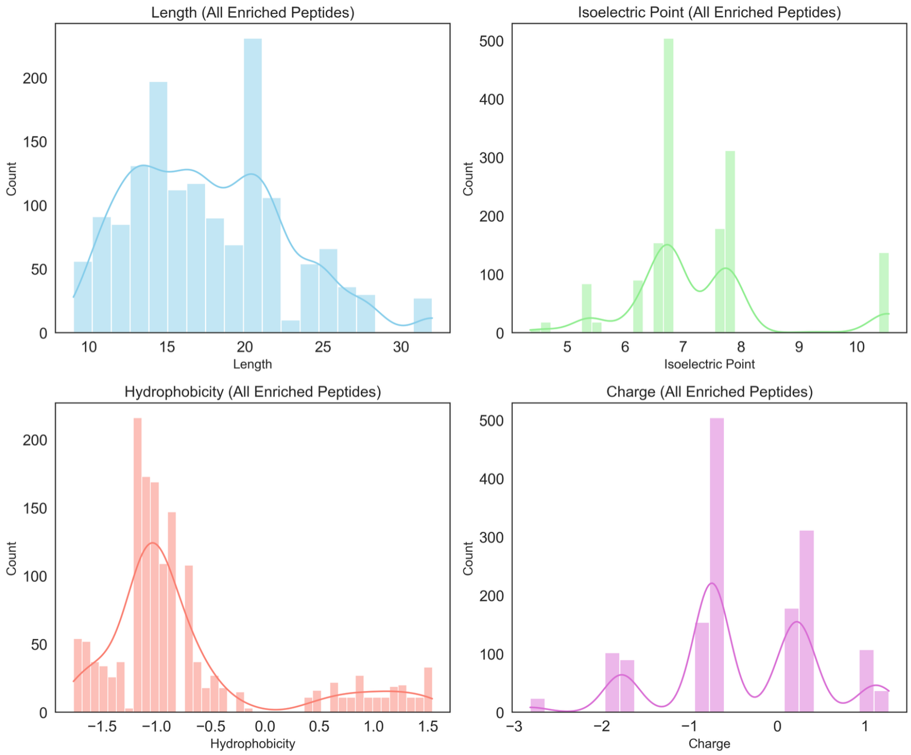

Figure S2. Distribution of physicochemical properties for all Cu^{2+}-IMAC enriched peptides. Histograms show peptide length, isoelectric point, hydrophobicity, and net charge distributions for the complete population of enriched peptides from both digestion schemes. Overlaid curves show kernel density estimates. The enriched population demonstrates narrow length distributions centered around 8-12 amino acids, neutral isoelectric points (~7), moderate hydrophobicity, and predominantly positive net charges.

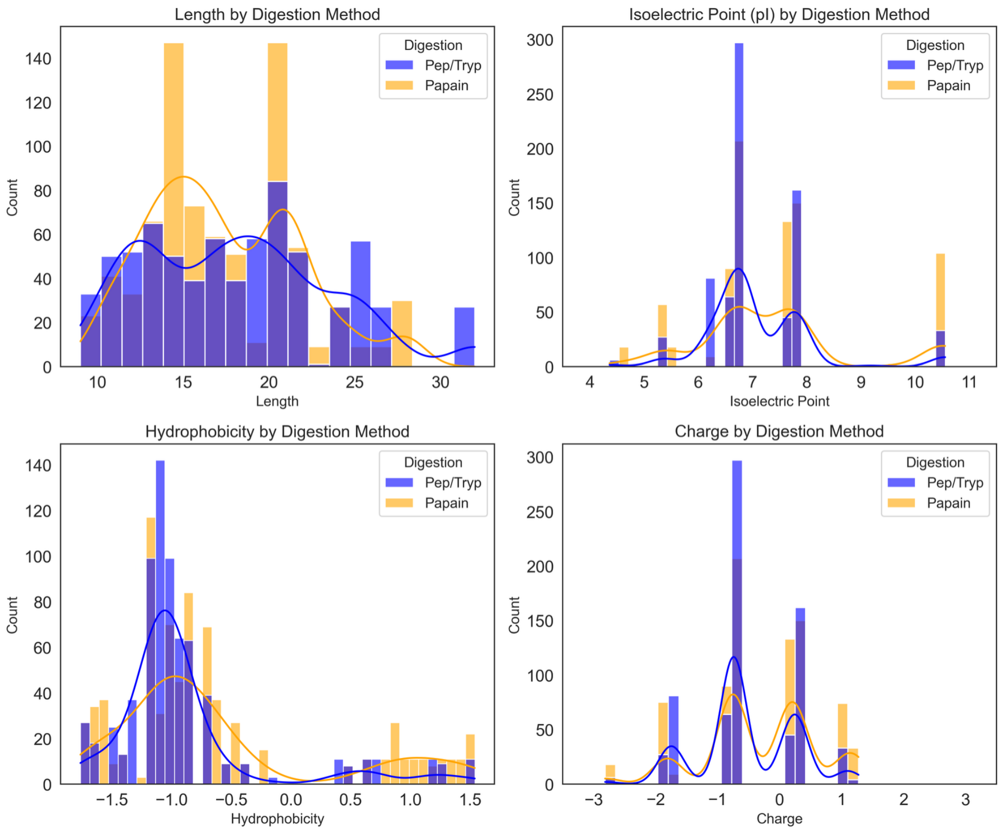

Figure S3. Comparison of physicochemical property distributions between digestion methods. Histograms show peptide length, isoelectric point, hydrophobicity, and net charge distributions for papain-digested (orange) and Pep/Tryp-digested (blue) peptides after Cu^{2+}-IMAC enrichment. Overlaid curves show kernel density estimates. Despite identical enrichment procedures, digestion methods produce statistically significant differences in peptide populations (Kolmogorov-Smirnov test, $p < 0.05$), though practical differences are modest, demonstrating that enzymatic specificity influences which peptide populations become accessible for metal affinity retention.

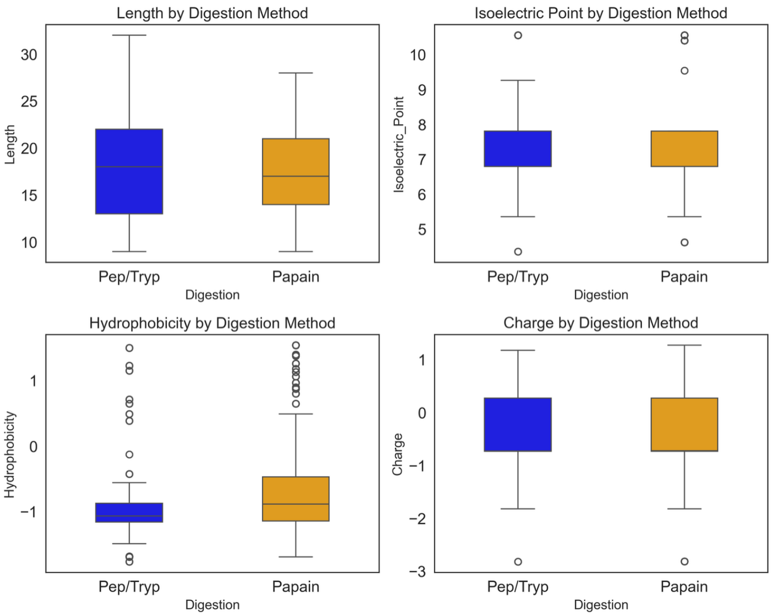

Figure S4. Physicochemical analysis reveals enriched peptides have a wide length distribution (10-25 amino acids), neutral isoelectric points (~7), moderate hydrophobicity, and net positive charges, indicating selection for optimal metal-coordinating architectures rather than simple electrostatic attraction.

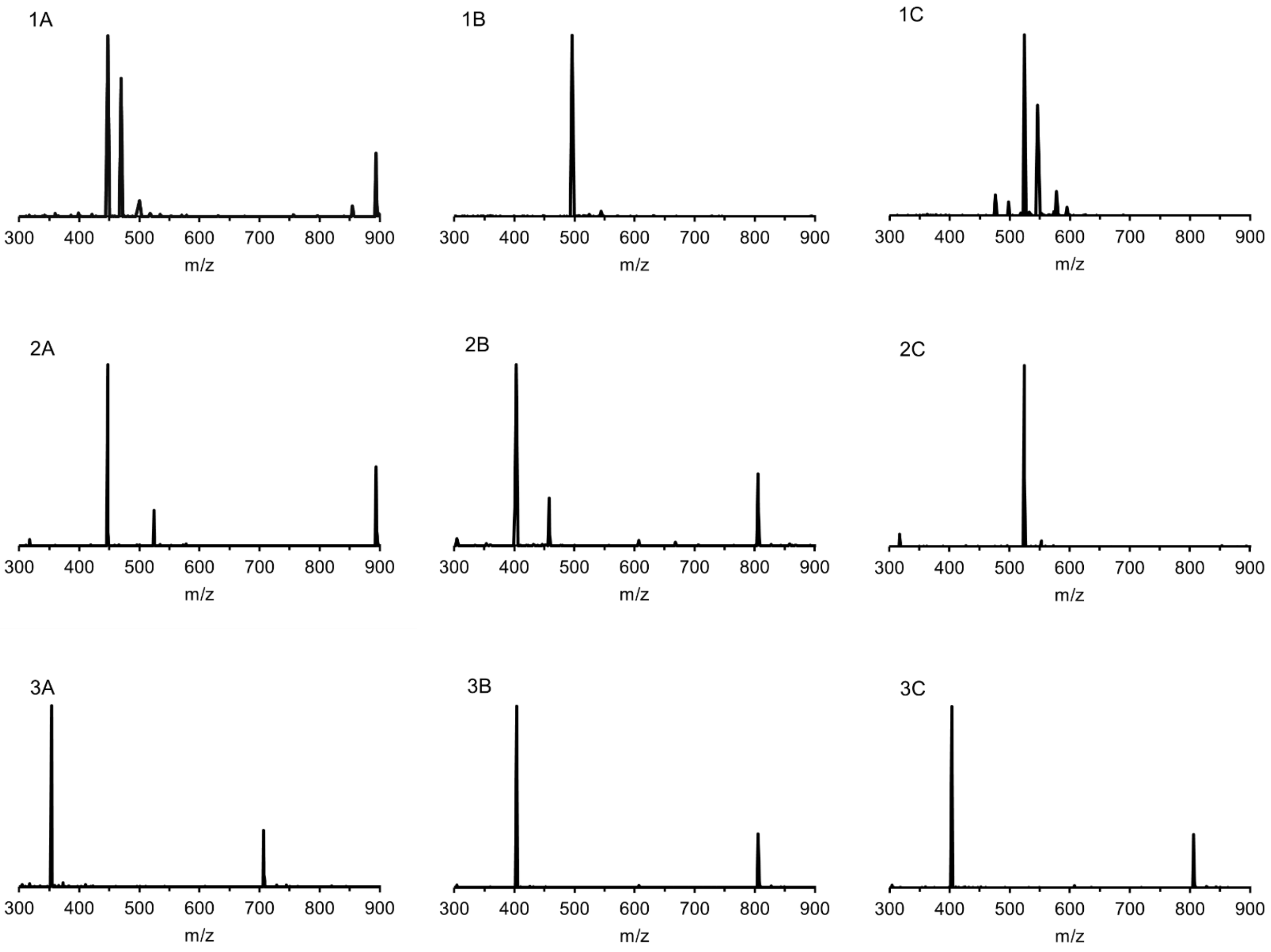

Figure S5. Nine peptides were synthesized by solid-phase peptide synthesis and verified via ESI-MS.

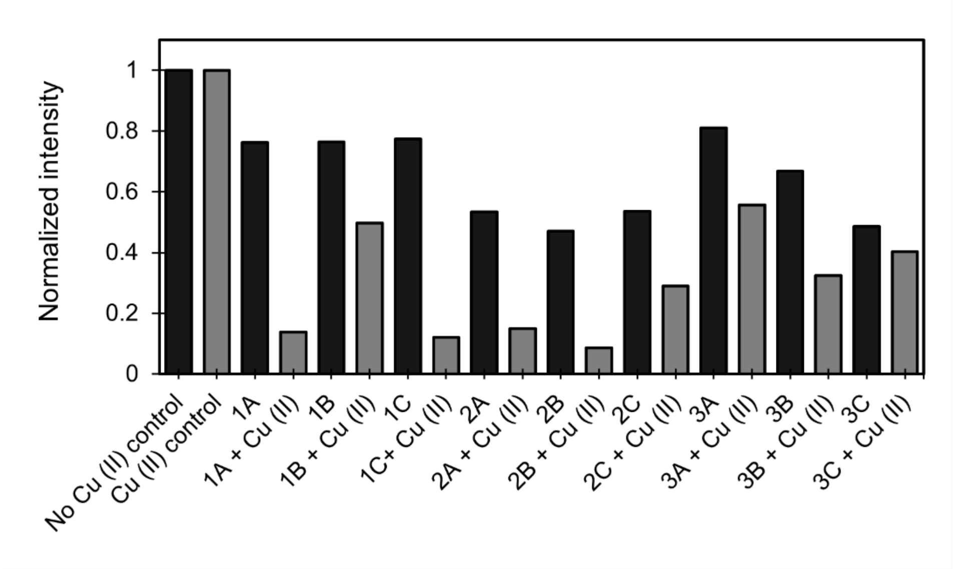

Figure S6. Fluorescent 7-OH-CCA assay (ex. 338 nm, em. 450 nm) measures the redox protection from Cu(II)-induced HO• generation of solutions containing 40 μM peptide in the absence (black bar) or presence of 10 μM $CuSO_4$ (grey bar). Data shown are normalized to either a no Cu(II) control (phosphate buffer) in the case of peptide alone, or a Cu(II) control in the case of peptide + Cu(II).

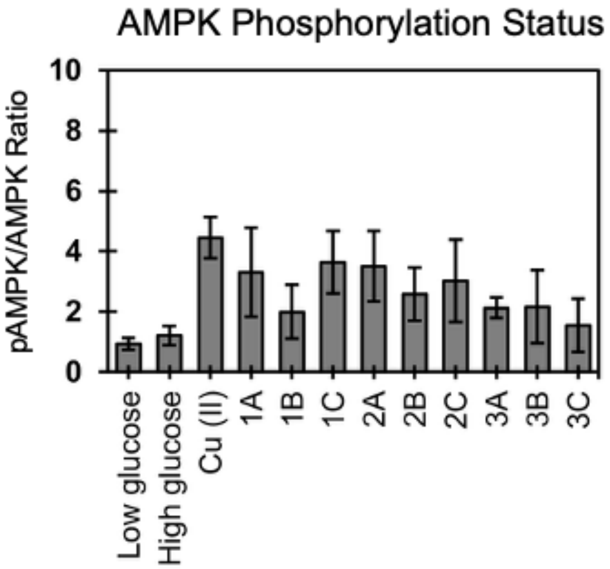

Figure S7. Densitometry of Western blots (n=3) for AMPK phosphorylation, as measured by ratio of phosphorylated AMPK to total AMPK.

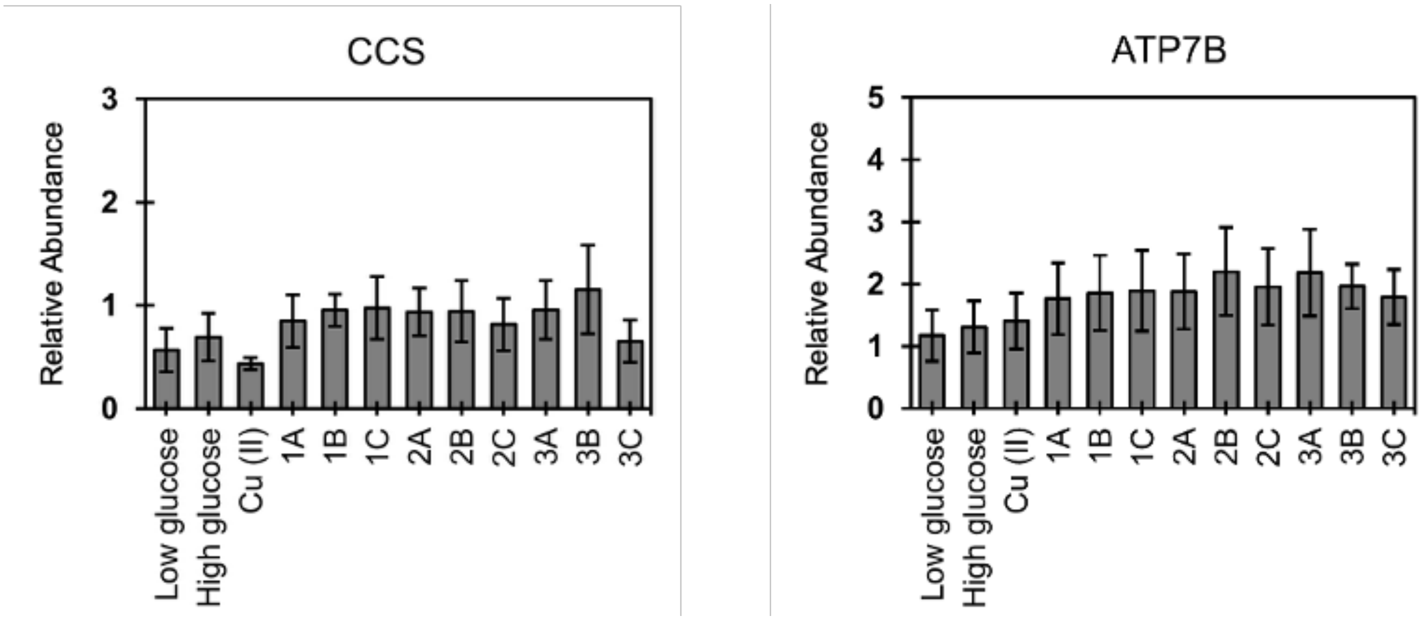

Figure S8. Densitometry of Western blots (*n=3*) for CCS and ATP7B**.**
